# Study on an Assembly Prediction Method of RV Reducer Based on IGWO Algorithm and SVR Model

**DOI:** 10.3390/s23010366

**Published:** 2022-12-29

**Authors:** Shousong Jin, Mengyi Cao, Qiancheng Qian, Guo Zhang, Yaliang Wang

**Affiliations:** College of Mechanical Engineering, Zhejiang University of Technology, Hangzhou 310023, China

**Keywords:** RV reducer, support vector regression, grey wolf optimization

## Abstract

This paper proposes a new method for predicting rotation error based on improved grey wolf–optimized support vector regression (IGWO-SVR), because the existing rotation error research methods cannot meet the production beat and product quality requirements of enterprises, because of the disadvantages of its being time-consuming and having poor calculation accuracy. First, the grey wolf algorithm is improved based on the optimal Latin hypercube sampling initialization, nonlinear convergence factor, and dynamic weights to improve its accuracy in optimizing the parameters of the support vector regression (SVR) model. Then, the IGWO-SVR prediction model between the manufacturing error of critical parts and the rotation error is established with the RV-40E reducer as a case. The results show that the improved grey wolf algorithm shows better parameter optimization performance, and the IGWO-SVR method shows better prediction performance than the existing back propagation (BP) neural network and BP neural network optimized by the sparrow search algorithm rotation error prediction methods, as well as the SVR models optimized by particle swarm algorithm and grey wolf algorithm. The mean squared error of IGWO-SVR model is 0.026, the running time is 7.843 s, and the maximum relative error is 13.5%, which can meet the requirements of production beat and product quality. Therefore, the IGWO-SVR method can be well applied to the rotate vector (RV) reducer parts-matching model to improve product quality and reduce rework rate and cost.

## 1. Introduction

With the continuous progress of digitalization, artificial intelligence, and smart production, rotate vector (RV) reducers, which are the critical constituents of industrial robots, have been mass-produced and widely used. However, limited to equipment precision and manufacturing capability, many RV reducer companies want to improve assembly precision and reduce scrap through parts matching. Thus, many scholars are devoted to researching various fast and accurate RV reducer assembly prediction methods and related contents to meet the enterprise production beat and product quality requirements. The key indicators to evaluate the quality of RV reducer are transmission power, torsional moment, transmission accuracy, service life, etc. [1], among which the indicator with a great impact on the transmission accuracy of RV-40E reducers is mainly the rotation error, the size of which is determined by the quality of each component in the RV-40E reducer drive chain and the quality of the assembly work [2].

Regarding the calculation of the rotation error, many studies have been conducted by domestic and foreign scholars; for example, Blanche [3] proposed a purely geometric method to determine the transmission error of the single cycloid of the planetary reducer. Takeshi Ishida [4] proposed a spring equivalence model to qualitatively analyze the RV rotation error by numerical analysis to establish accurate modeling for the RV reducer rotation error. Yinghui Zhang [5] carried out the simulation calculation of dynamic rotation error by establishing a virtual prototype model of RV reducers. Xiaotao Tong [6] proposed a back propagation (BP) neural network-based dynamic rotation error prediction method for RV reducers to achieve the prediction of the rotation error of RV reducers by including five critical factors of manufacturing errors in the second-stage cycloid pin-wheel transmission mechanism. Houzhen Sun [7] established a BP neural network transmission-error prediction model based on the sparrow search algorithm for back propagation (SSA-BP) and selected key dimensional parameters of assembled parts as influencing factors to achieve the prediction of key quality characteristics before assembly. These research results can calculate the RV reducer rotation error to a certain extent, but there are some defects, such as poor calculation accuracy and long operation time, that come up when used for actual assembly. For example, the calculation process of the pure geometric method is relatively cumbersome, and it does not take into account the actual part machining error and assembly error. Additionally, the mathematical analysis method of the equivalent model has a large gap between the final modeling results and the actual situation owing to massively simplified processing in the modeling process. Further, the model construction of the virtual prototype analysis method is complicated and time-consuming. Although the BP neural network prediction method can achieve the fast prediction of rotation errors, it needs massive sample data to obtain better prediction accuracy because of the complex internal structure. The existing research methods of rotation error are difficult to be used in practical assembly because of the disadvantages, such as being time-consuming and having poor calculation accuracy. Therefore, to address the abovementioned disadvantages of the existing research methods for rotation errors, this paper will establish a new prediction method for rotation errors to achieve fast and accurate predictions.

### 1.1. Literature Review

The problem of rotation error prediction is characterized by multifactor coupling, high-dimensional nonlinearity, and a limited number of samples, which makes the support vector regression (SVR) in machine learning have certain application advantages relative to the already-existing BP and SSA-BP neural network rotation error prediction methods. The SVR method in machine learning is characterized by strong generalizability and simple structure in solving small-sample high-dimensional nonlinear problems [8], and it is used in many fields. For example, Balogun [9] solved the spatial prediction problem of landslide sensitivity by using the SVR model combined with grey wolf optimization (GWO) algorithm, bat algorithm, and cuckoo optimization algorithm. Zichen Zhang [10] solved the power load forecasting problem by using the SVR model combined with GWO algorithm. Peng [11] solved the pipeline corrosion rate prediction problem by combining the SVR model with principal component analysis and the chaotic particle swarm optimization algorithm. Therefore, to address the shortcomings of existing rotation error research methods and the performance of the SVR method in solving high-dimensional nonlinear, small-sample problems, this paper will use the SVR method to establish a prediction model for rotation errors.

Although the modeling process is simple, the values of the penalty factor and the kernel function parameter in the SVR model have a significant impact on the model’s prediction performance [12,13]. Therefore, in order to use the SVR model to achieve better predictions for rotation errors, the problem of parameter optimization in the SVR model first needs to be solved. Currently, population intelligence algorithms and their improved versions are becoming increasingly popular in solving different optimization problems and are widely used in many fields [14]. For example, for the dynamic economic emission dispatch problems, Zhifeng Liu [15] proposed a novel solving approach based on the enhanced moth-flame optimization algorithm, which effectively reduced fuel costs as well as the pollutant emissions of power generation systems; Lingling Li [16] proposed an improved tunicate swarm algorithm for optimizing fuel costs and pollutant emissions, which optimized the optimal dynamic scheduling scheme. For feature-selection problems, Tubishat [17] proposed a dynamic salp swarm algorithm (DSSA), and the improvements significantly enhanced its performance in solving feature-selection problems; Hongliang Zhang [18] used chaotic initialization and differential evolution to improve the sparrow search algorithm (SSA) as a way to increase the convergence speed of the algorithm, which achieved good optimization results in the feature-selection problem. In the field of mechanical engineering optimization problems, population intelligence algorithms solve a wide variety of optimization problems [19]. Abderazek [20] proposed a queuing search algorithm to optimize the main parameters of the spur gear. Yaliang Wang [21] proposed a cellular differential evolutionary algorithm with double-stage external population leading to solve the optimal design of cycloid reducer parameters. Because of the powerful optimization capability of population intelligence algorithms, domestic and foreign scholars usually also use intelligent algorithms such as particle swarm optimization (PSO) and GWO algorithms to optimize the parameters in the SVR model [22,23].

The GWO algorithm is a novel heuristic algorithm proposed by Mirjalili et al. in 2014, which has the advantages of fast convergence speed and high solution accuracy compared with other intelligent algorithms [24]. However, the algorithm itself has the drawbacks of poor initial population diversity, slow convergence speed at the later stage, and easy-to-fall-into local optimum. Additionally, for these drawbacks, various improvement methods have been proposed and successfully applied by domestic and foreign scholars [25,26]. Although the GWO algorithm has stronger convergence performance than other traditional intelligent algorithms, it does not have a great advantage in the parameter optimization problem, so in order to further improve the performance of the algorithm in optimizing parameters, this paper proposes an improved grey wolf optimization (IGWO) algorithm based on the initialization of the optimal Latin hypercube sampling (OLHS) method, the nonlinear convergence factor, and the dynamic weight. In this study, the optimization capability of the proposed IGWO algorithm is compared with the GWO algorithm, the PSO algorithm, and the advanced SSA algorithm on six test functions, and the performance of the IGWO algorithm in solving the SVR model parameters optimization problem is verified by an actual case.

### 1.2. Major Contributions

In order to solve the problem that the current research method of rotation error is difficult to apply to the actual assembly process of RV reducer owing to its shortcomings, such as its being time-consuming and having poor calculation accuracy, and given the high-dimensional, nonlinear, and small-sample-size characteristics of the rotation error prediction problem, this paper proposes a method of rotation error prediction based on improved grey wolf–optimized support vector regression (IGWO-SVR). In addition, in order to improve the prediction accuracy of the SVR model, this paper improves the initialization population, the convergence factor, and the head wolves’ weights of the GWO algorithm to improve its accuracy for SVR model parameter optimization, and its optimization performance is verified by test functions. Finally, the parameter optimization ability of the IGWO algorithm and the prediction performance of the IGWO-SVR method are verified by using a practical case. The main contributions of this study are as follows:

- An improved grey wolf optimization algorithm is proposed, with three improvements:
  1. Improving their initialized populations through the optimal Latin hypercube sampling idea as a way to increase initial population diversity.
  2. Improving the convergence factor by the cosine nonlinear function, which improves the global search ability in the early stage and the convergence speed in the later stage of the algorithm.
  3. Improving the speed of convergence of this algorithm to the optimal solution through the improvement of the dynamic weighting strategy.
- Establish a new rotation error prediction method based on the IGWO algorithm and the SVR model to achieve fast and accurate predictions of rotation errors.
- The IGWO-SVR method shows better prediction performance relative to other rotation error prediction methods, and the IGWO algorithm also shows good parameter optimization performance, as verified by the RV reducer example.

## 2. Structural Principle and Rotation Error of RV Reducer

### 2.1. Structural Principle Analysis of RV Reducer

**Figure 1:**
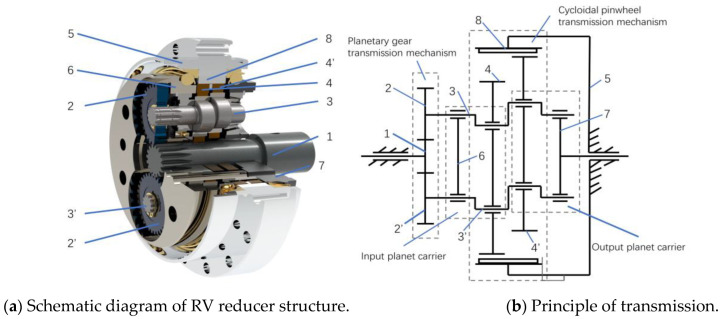
Structural schematic diagram of RV reducer. 1. Input shaft; 2. Planetary wheel; 3. Crankshaft; 4. Cycloid wheel; 5. Needle tooth shell; 6. Flange; 7. Planet carrier; 8. Needle gear.

There are various models of RV reducers, and this article is mainly about the RV-40E reducer. The RV-40E reducer is constituted primarily of a planetary wheel, a crankshaft, a cycloid wheel, a flange, a needle gear, etc. Figure 1a shows a schematic diagram of its structure. Rotation error, which is the critical quality characteristic of the RV reducer, can be affected by the processing error of each component of the transmission chain, the modification amount of contact gear tooth profile, assembly clearance, elastic-plastic deformation, etc. [27]. Additionally, its size refers to the difference between the theoretical output angle and the actual output angle.

The transmission of the RV reducer is divided into two stages: the first-stage involute planetary gear transmission and the second-stage cycloid transmission. Figure 1b shows the principle of the RV reducer transmission. Because the first-stage transmission system is far from the output end and because the transmission ratio of the second-stage transmission is 3~25 times that of the first-stage transmission, the impact of the first-stage involute gear planetary transmission system on the overall rotation error of the assembly will be greatly reduced. Second, because the influence of the second-stage cycloid pin-wheel transmission on the transmission error is directly reflected on the output shaft, the effect on the transmission accuracy of the RV reducer depends mainly on the second-stage rotation error [28]. In conclusion, this paper considers only the second-stage cycloid pin-wheel transmission system to establish the rotation error prediction model and ignores the first-stage transmission.

### 2.2. Analysis of Influencing Factors of Rotation Error

There are many manufacturing errors that affect the rotation error in the second-stage transmission system, and it is difficult to study the manufacturing errors of the parts one by one. Therefore, this paper uses the Taylor series expansion principle to conduct a sensitivity analysis of manufacturing errors to identify the primary influencing elements from the manufacturing errors in the second-stage cycloidal pin-wheel transmission. Additionally, parameters for each influencing factor are noted as $\;\theta =\left[\theta_{1},\theta_{2},\cdots ,\theta_{n}\right]$. According to the principle of sensitivity analysis, the rotation error model is defined in Equation (1):

(1) $$\varphi =\varphi \left(\theta_{1},\theta_{2},\cdots ,\theta_{n}\right)$$

Using the Taylor series expansion principle, the sensitivity index of each error can be defined in the Equation (2):

(2) $$S_{i}=\frac{\partial \varphi /\partial \theta_{i}}{\partial \varphi /\partial \theta_{0}}\;i=1,2,3\cdots n\;$$

where $\partial \varphi /\partial \theta_{0}$ is the reference factor and the crank-bearing clearance is chosen as the reference in this paper.

Table 1 shows the sensitivity analysis results of eight key manufacturing errors in the second-stage cycloidal pin-wheel transmission. According to Table 1, this paper selects five factors, including the cycloid gear isometric modification error ($\theta_{1}$), the radius error of needle tooth center circle ($\theta_{2}$), the cycloid gear shift modification error ($\theta_{3}$), the needle tooth radius error ($\theta_{4}$), and the crankshaft eccentricity error ($\theta_{5}$), to establish the rotation error prediction model. Although the sensitivity index of the crankshaft eccentricity error is very small, the crank-bearing clearance error that can be caused by the crankshaft eccentricity error has a great influence on rotation error; therefore, the crankshaft eccentricity error is also considered to establish the prediction model.

## 3. The Improvement of the GWO Algorithm

### 3.1. GWO Algorithm

The GWO was proposed in 2014 by Mirjalili [24] as a novel heuristic group intelligence method, which is based on the principle of simulating the hierarchical mechanism and hunting mode of the grey wolf pack. The grey wolf pack is generally divided into four classes, and the top-three wolves with the best adaptability in the pack are divided into *wolfα*, *wolfβ*, and *wolfγ*, and the rest are *ω* wolves. Its hunting process is mainly that *wolfα*, *wolfβ*, and *wolfγ* lead other *ω* wolves to continuously search, surround, and attack prey. Additionally, update the *wolfα*, *wolfβ*, and *wolfγ* of each iteration during the hunting process.

Step 1: surrounding the prey. The wolf pack *ω* is constantly updated according to the prey’s position and its distance from the prey, thus gradually approaching the prey. The mathematical formula is as follows:

(3) $$D=\left|C\cdot X_{p}\left(t\right)-X\left(t\right)\right|$$

(4) $$X\left(t+1\right)=X_{p}\left(t\right)–A\cdot D$$

where D denotes the distance between *ω* wolf and prey, t denotes the present iterations, $X_{p}\left(t\right)$ denotes the location of prey at tth iterations, X(t) denotes the location vector of grey wolf in tth iterations, and X(t+1) denotes the updated position of the ω wolves in (t+1)th iteration. Finally, C and A are the random coefficients, which can be formulated as follows:

(5) $$A=2\alpha \cdot r_{1}-\alpha$$

(6) $$C=2r_{2}$$

(7) $$\alpha =2-2\frac{t}{t_{max}}$$

where $r_{1}$, $r_{2}$ denote the random vectors of [0, 1] interval, t denotes the present iterations, $t_{max}$ denotes the maximum iterations, and α denotes a converging factor.

**Figure 2:**
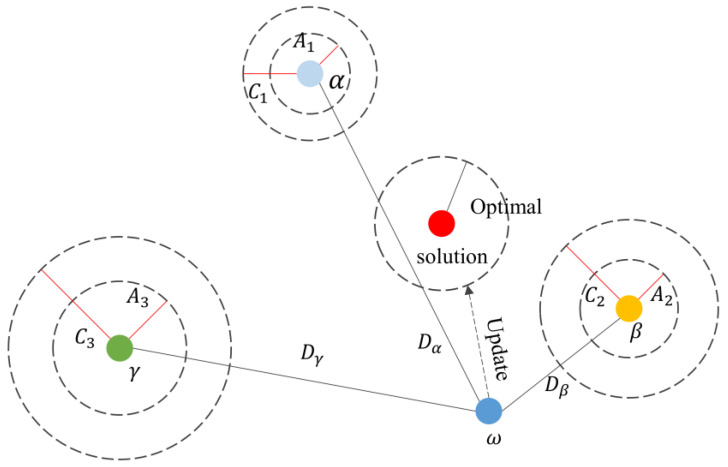
Schematic diagram of grey wolf location update.

Step 2: hunting. The hunting behavior is completed under the leadership of the *wolfα*, *wolfβ*, and *wolfγ*. The leading of *ω* wolves by leaders depends mainly on the constant A and the distance D between *ω* wolf and prey. Figure 2 shows a schematic diagram of a *ω* wolf position update, which can be formulated as follows:

(8) $$\{\begin{array}{c} D_{\alpha }=\left|C\cdot X_{\alpha }-X\left(t\right)\right|\\ D_{\beta }=\left|C\cdot X_{\beta }-X\left(t\right)\right|\\ D_{\gamma }=\left|C\cdot X_{\gamma }-X\left(t\right)\right| \end{array}$$

(9) $$\{\begin{array}{c} X_{1}=X_{\alpha }-A_{1}\cdot D_{\alpha }\\ X_{2}=X_{\beta }-A_{2}\cdot D_{\beta }\\ X_{3}=X_{\gamma }-A_{3}\cdot D_{\gamma } \end{array}$$

(10) $$X\left(t+1\right)=\frac{X_{1}+X_{2}+X_{3}}{3}$$

where $D_{\alpha },D_{\beta }$ and $D_{\gamma }$ indicate the distance between the grey wolf and the prey; $X_{\alpha }$, $X_{\beta }$, and $X_{\gamma }\;$ indicate the position of the *wolfα*, *wolfβ*, and *wolfγ*, respectively; and X(t+1) indicates the updated location of the wolf pack ω in the (t+1)th iteration. The iterations are performed by the above method, and the optimal solution is obtained when the iteration times satisfy the termination condition.

### 3.2. Improved GWO Algorithm

The traditional GWO algorithm has some disadvantages: poor initial population diversity, slow convergence in the later stage, and easy-to-fall-into local optimization [25]. Additionally, the inaccuracy of the parameter optimization results of the GWO algorithm may result in poor forecast precision from the SVR model for the rotation error, thus failing to meet the requirements of the RV reducer assembly line. Hence, this article proposes an IGWO algorithm, based on the initialization of the OLHS method, the cosine nonlinear convergence factor, and dynamic weights to improve the accuracy of the algorithm’s parameter search.

#### 3.2.1. Wolf Pack Initialization by the OLHS Method

In intelligent optimization algorithms, the distribution of the initial population location influences the global search speed and the convergence accuracy of algorithms, and the uniform and diverse population distribution facilitates the optimization properties of the algorithm [29]. The traditional GWO algorithm uses a random method to generate the initial population, so it lacks diversity. In addition, the uneven distribution of the initial population may cause it to run into a topical optimization solution; that is, the initial range does not cover the location of the optimal solution, so the algorithm cannot find the global optimal solution.

The OLHS method is an improvement on the Latin hypercube sampling (LHS) method. The traditional LHS rule can ensure only that the projection is uniformly distributed on each coordinate axis, but the distribution in space is not uniform. Therefore, in the case of improving its space-filling property, in 1990 Johnson [30] proposed the minimax rule to improve the space-filling degree, which is one of the most widely used methods to evaluate sampling uniformity. However, the calculation scale of this method is very large, so Morris [31] proposed the OLHS method in 1995 on the basis of the scalar discriminant function, which not only guaranteed the characteristics of space-filling but also reduced the calculation scale. Its scalar discriminant function formula is as follows:

(11) $$\varphi_{q}\left(X\right)={\left(\overset{m}{\underset{i=1}{\sum }}J_{i}d_{i}^{-q}\right)}^{\frac{1}{q}}$$

where $d_{i},i=1,2,3\ldots \ldots m$ indicates the distance between all possible combinations of two points in the sampling matrix, X, $j_{i},i=1,2,3\ldots \ldots m$ indicates the number of pairs of points with distance $d_{i}$ in the sampling matrix *X*, and q indicates the mode norm of the space, which is a positive integer.

**Figure 3:**
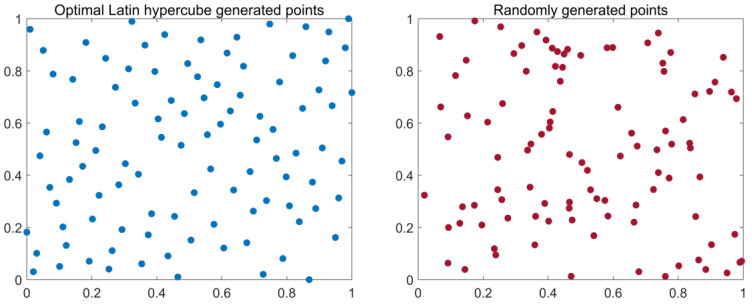
Comparison between generating point of OLHS and random generating point.

In this paper, the OLHS method with good space-filling property is used to initialize the distribution position of grey wolves. Figure 3 shows the different distributions of the initializations of the random method and the OLHS method when generating 100 grey wolf individuals between [0, 1] in two dimensions. According to Figure 3, it is obvious that the initialization of the OLHS method can result in a more uniform distribution of the initial population in the sample space, and the more uniform initial population can provide more information on the global optimal solution, which can improve the algorithm’s global search capability and convergence speed.

#### 3.2.2. Nonlinear Convergence Factor

The key problem of the traditional GWO algorithm is how to make a trade-off between the ability of global optimization and local optimization. Additionally, the global optimization capacity affects the stability and accuracy of the algorithm optimization, and the local optimization capacity affects the rate of the algorithm convergence. The traditional GWO algorithm balances the ability of global and local searches by the change in A, which is controlled by the linear variation of *α*. When |A|≤1, the algorithm tends to develop the capacity of local searches; when |A |>1, the algorithm tends to develop the capacity of global searches.

**Figure 4:**
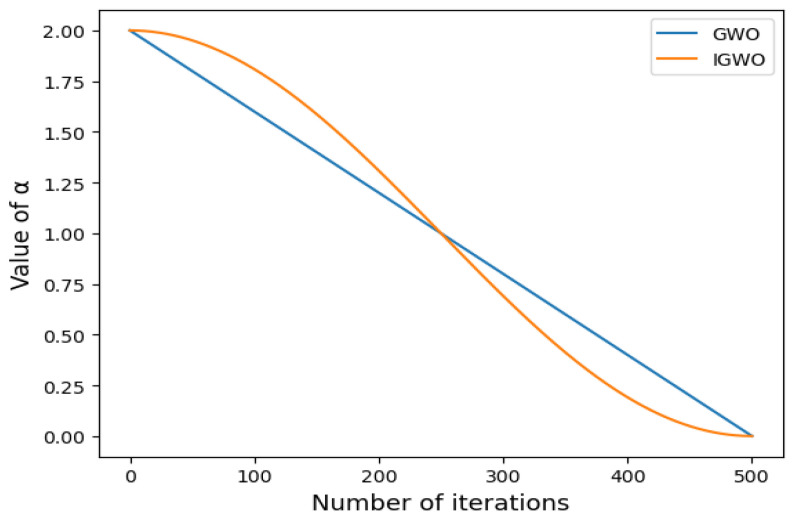
The decreasing process of the convergence factor.

The convergence factor parameter α  that is shown in Equation (5) is a linear, decreasing transformation that represents the convergence process from 2 to 0. However, the convergence of the algorithm is often a nonlinear process, so the linear decrement of α is difficult to use to control the convergence process of the algorithm, causing the algorithm to easily run into a local optimum [32]. The research of Yuxiang Hou [33] shows that the nonlinear transformation of parameter α can enhance the optimization capacity of the algorithm and avoid running into a local optimum. Therefore, this paper uses cosine nonlinear convergence parameter α to control the convergence process of the algorithm, and its formula is given by Equation (12):

(12) $$\alpha =\cos\;\left(\frac{t}{t_{max}}\pi \right)+1$$

where t indicates the iterations, $t_{max}$ indicates the maximum iterations, and α indicates the convergence factor. The convergence processes of α for the IGWO algorithm and traditional GWO algorithm are shown in Figure 4.

According to Figure 4, the α of the GWO algorithm linearly decreases, which does not apply to the nonlinear convergence process of the algorithm. The α of the IGWO algorithm is nonlinear and normally decreases, and during the first half of the iteration, the convergence factor decreases more slowly compared with the traditional GWO algorithm, thus improving the global search ability of the algorithm and avoiding the algorithm’s falling into the local optimum in the early stage. During the second half of the iteration, the decreasing speed of the convergence factor is accelerated, thus improving the convergence speed and accuracy of the local search of the algorithm. In summary, the cosine nonlinear improvement of the convergence factor can better equilibrate the capabilities of the global optimization and the local optimization of the algorithm.

#### 3.2.3. Weight-Based Grey Wolf Position Update

In the GWO algorithm, the leading weights of *wolfα*, *wolfβ*, and *wolfγ* are the same, which leads to a slow convergence of the algorithm. In addition, many scholars have verified that a better new grey wolf pack can be generated by weighted fitness or weighted distance, thus accelerating the convergence speed of the algorithm. In this paper, the weight is dynamically adjusted according to the fitness and distance, and the position update formula in Equation (10) is transformed into Equation (14). The mathematical expressions of weight and position update formula are as follows:

(13) $$\left\{ \begin{array}{c} W_{\alpha }^{1}=\frac{f_{\alpha }+f_{\beta }+f_{\gamma }}{f_{\alpha }},\;W_{\beta }^{1}=\frac{f_{\alpha }+f_{\beta }+f_{\gamma }}{f_{\beta }},\;W_{\gamma }^{1}=\frac{f_{\alpha }+f_{\beta }+f_{\gamma }}{f_{\gamma }}\\ W_{\alpha }^{2}=\frac{\left|X_{1}\right|}{\left|X_{1}+X_{2}+X_{3}\right|},\;W_{\beta }^{2}=\frac{\left|X_{2}\right|}{\left|X_{1}+X_{2}+X_{3}\right|},\;W_{\gamma }^{2}=\frac{\left|X_{3}\right|}{\left|X_{1}+X_{2}+X_{3}\right|}\\ W_{1}=\frac{\sqrt{W_{\alpha }^{1}*W_{\alpha }^{2}}}{\sqrt{W_{\alpha }^{1}*W_{\alpha }^{2}}+\sqrt{W_{\beta }^{1}*W_{\beta }^{2}}+\sqrt{W_{\gamma }^{1}*W_{\gamma }^{2}}}\\ W_{2}=\frac{\sqrt{W_{\beta }^{1}*W_{\beta }^{2}}}{\sqrt{W_{\alpha }^{1}*W_{\alpha }^{2}}+\sqrt{W_{\beta }^{1}*W_{\beta }^{2}}+\sqrt{W_{\gamma }^{1}*W_{\gamma }^{2}}}\\ W_{3}=\frac{\sqrt{W_{\gamma }^{1}*W_{\gamma }^{2}}}{\sqrt{W_{\alpha }^{1}*W_{\alpha }^{2}}+\sqrt{W_{\beta }^{1}*W_{\beta }^{2}}+\sqrt{W_{\gamma }^{1}*W_{\gamma }^{2}}} \end{array}\right.$$

(14) $$X\left(t+1\right)=W_{1}X_{1}+W_{2}X_{2}+W_{3}X_{3}$$

where t indicates the current iteration number; $f_{\alpha },\;f_{\beta }$, and $f_{\gamma }$ are the current fitness values of *wolfα*, *wolfβ*, and *wolfγ*, respectively; and X(t+1) represents the position of the (t+1)th iteration *ω* wolf.

#### 3.2.4. Validation of IGWO Algorithm

**Figure 5:**
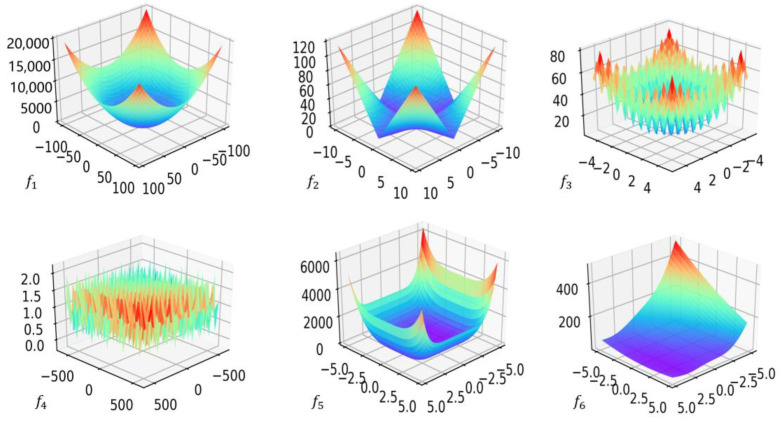
Three-dimensional graphs of functions.

In the literature, an article [34] chose six functions to test the properties of the IGWO algorithm, among which $f_{1}$ and $f_{2}$ are single-peaked functionns, $f_{3}$ and $\;f_{4}$ are multipeaked functions, and $f_{5}$ and $f_{6}$ are fixed-dimension multipeaked functions, as shown in Table 2 and Figure 5. Additionally, it compared the IGWO algorithm with the PSO algorithm, the SSA algorithm, and the traditional GWO algorithm to better demonstrate the first’s seeking performance. To ensure fairness and effectiveness, the maximum iteration number and the initial population size of algorithms were uniformly set to 500 and 30, $c_{1}$ = 2, $c_{2}$ = 2, ω = 0.75 for the PSO algorithm, q = 10 for the IGWO algorithm, and $R_{2}$ = 0.8, ST = 0.8, percent  = 0.2 for the SSA algorithm.

**Figure 6:**
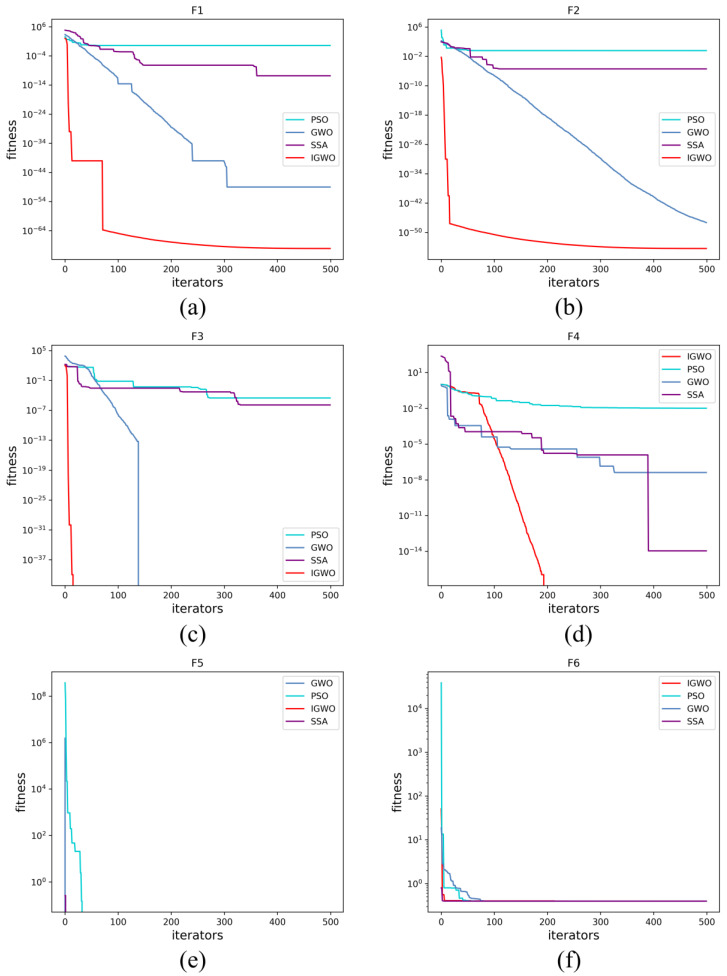
The convergence curves of algorithms on test functions. (**a**) $f_{1}$ function; (**b**) $f_{2}$ function; (**c**) $f_{3}$ function; (**d**) $f_{4}$ function; (**e**) $f_{5}$ function; (**f**) $f_{6}$ function.

The average and the standard deviation of the 20 optimization results are used as the criteria to reflect the optimization performance of the algorithms. Table 3 and Figure 6 show the optimization results and convergence curves. The results show that for single-peak functions $f_{1}$ and $f_{2}$, the mean of the IGWO algorithm is more similar to the real optimal solution and that the algorithm converges faster compared with the PSO, GWO, and SSA algorithms. For multipeaked functions $f_{3}$ and $f_{4}$, the IGWO algorithm shows good optimization performance, and there is no error between its optimization results and the real results, whereas all other algorithms have some errors. For the fixed-dimensional multipeaked functions $f_{5}$ and $f_{6}$, the convergence results of all four algorithms are accurate. Therefore, the IGWO algorithm has better function optimization performance and better convergence speed and accuracy than the PSO, GWO, and SSA algorithms, which indicates that the enhancement of the algorithm in this article is efficient.

## 4. Rotation Error Prediction Model Based on IGWO-SVR

### 4.1. SVR Model

Support vector regression is generalized from support vector machines and applied to solve regression prediction problems [35]. For the problem of predicting the rotation error of the RV reducer, cycloid gear isometric modification error ($\theta_{1}$), needle tooth center circle radius error ($\theta_{2}$), cycloid gear shift modification error ($\theta_{3}$), needle tooth radius error ($\theta_{4}$), and crankshaft eccentricity error ($\theta_{5}$) are composed of input variables, and the rotation error is taken as the output variable. Thus, the sample can be formulated as follows:

(15) $$x_{i}=\left[\theta_{1},\;\theta_{2},\;\theta_{3},\theta_{4},\;\theta_{5}\right]$$

(16) $$X={\left[x_{1},\;x_{2},\;x_{3},\;x_{4},\ldots ,x_{n}\right]}^{T}$$

(17) $$Y={\left[Y_{1},\;Y_{2},\;Y_{3},\;Y_{4},\ldots ,Y_{n}\right]}^{T}$$

where *n* denotes the number of samples, $x_{i}$ represents the input variables, X represents an array of input factor variables, and Y denotes an actual value array of sample data rotation errors.

According to the sample data, if there is a functional relationship between the rotation error and the five key manufacturing errors above. The SVR model is shown in Equation (18):

(18) $$f\left(x\right)=\omega^{T}\varphi \left(x\right)+b$$

where x is the input vector, f(x) denotes the predicted value of rotation error, φ(x) is the mapping relationship function, and b and ω are the deviation term and the weight vector, respectively.

By introducing an insensitive loss function and minimizing structural risk, Equation (18) can be transformed as follows:

(19) $$min\frac{1}{2}{\left|\left|\omega \right|\right|}^{2}+C\overset{n}{\underset{i=1}{\sum }}\left({\xi \;}_{i}^{-}+{\xi \;}_{i}^{+}\right)$$

(20) $$s.t.\{\begin{array}{c} Y_{i}-\left(\omega^{T}\varphi \left(x_{i}\right)+b\right)\leq \varepsilon +{\xi \;}_{i}^{+}\\ \left(\omega^{T}\varphi \left(x_{i}\right)+b\right)-Y_{i}\leq \varepsilon +{\xi \;}_{i}^{-}\\ {\xi \;}_{i}^{-},{\xi \;}_{i}^{+}\geq 0,\;i=1,2,\cdot \cdot \cdot ,n \end{array}$$

where ${\left|\left|\omega \right|\right|}^{2}$ is the regular term, ε is the parameter of the insensitive penalty function, ${\xi \;}_{i}^{-}$ and ${\xi \;}_{i}^{+}$ are slack variables, $x_{i}$ is the input variable, $Y_{i}$ is the actual value of the rotation error, and C is a penalty factor.

By introducing Lagrange multipliers ${\alpha \;}_{i}^{+},{\;\alpha \;}_{i}^{-}$ and kernel functions, (19) and (20) can be transformed into their dual form problem, as shown in Equations (21) and (22):

(21) $$max\left[\frac{1}{2}\sum_{i=1}^{n}\sum_{j=1}^{n}\left(\alpha_{i}^{+}-\alpha_{i}^{-}\right)\left(\alpha_{j}^{+}-\alpha_{j}^{-}\right)K\left(x,x_{i}\right)-\varepsilon \sum_{i=1}^{n}\left(\alpha_{i}^{+}+\alpha_{i}^{-}\right)+\sum_{i=1}^{n}\left(\alpha_{i}^{+}-\alpha_{i}^{-}\right)y_{i}\right]$$

(22) $$s.t.\{\begin{array}{c} \overset{n}{\underset{i=1}{\sum }}\left({\alpha \;}_{i}^{+}-{\alpha \;}_{i}^{-}\right)=0\\ 0\leq {\alpha \;}_{i}^{+},{\alpha \;}_{i}^{-}\leq C \end{array}$$

where ${\alpha \;}_{i}^{+},{\alpha \;}_{i}^{-}$ are Lagrange multipliers and $K\left(x,x_{i}\right)$ is a Gauss radial basis kernel function. The formula is given by Equation (23):

(23) $$K\left(x,x_{i}\right)=exp\left(-\frac{{\left|\left|x-x_{i}\right|\right|}^{2}}{2\sigma^{2}}\right)=\exp\left(-g{\left|\left|x-x_{i}\right|\right|}^{2}\right)$$

where σ denotes the width of the function and g represents the parameter of the kernel.

Finally, through the Karush–Kuhn–Tucker condition, the mathematical expression of the final SVR model is shown in Equation (24):

(24) $$f\left(x\right)=\overset{n}{\underset{i=1}{\sum }}\left({\alpha \;}_{i}^{+}-{\alpha \;}_{i}^{-}\right)K\left(x,x_{i}\right)+b$$

### 4.2. Process of Building Rotation Error Prediction Model

The prediction of the rotation error is a typical high-dimensional nonlinear problem. By the SVR method, the kernel function throws the sample data from the low dimensional space to the relatively higher dimensional space to solve the problem.

**Figure 7:**
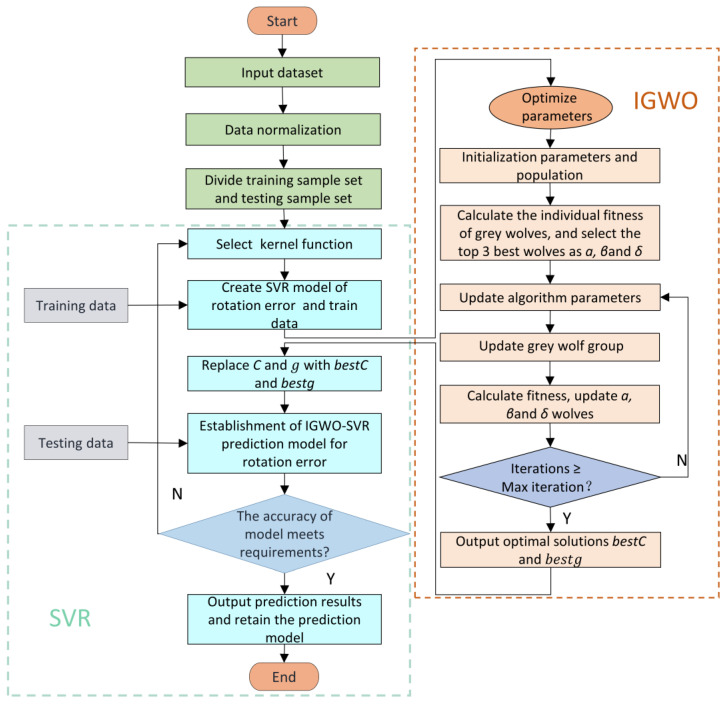
Flowchart of the IGWO-SVR method.

The prediction accuracy of rotation errors based on SVR is significantly affected by C and g, as shown in Equations (23) and (24). First, the penalty factor C represents the penalty on the sample points and beyond the ±ε pipe, and the magnitude of this value has an impact on the stability as well as the complexity of the SVR prediction model. In addition, the kernel function g represents the correlation between the sample points and beyond the ±ε tube. The larger the g value is, the stronger the correlation between these points is, but the accuracy of the rotation error prediction model is difficult to guarantee. On the contrary, the smaller the g value is, the looser the correlation between these points is, resulting in a more complex SVR model and the poorer universalization capacity of the model. Therefore, taking the appropriate *C* and g can enhance the precision of the SVR model for predicting rotation errors. To enhance the prediction precision of the SVR model for RV reducer rotation errors, this article uses the abovementioned IGWO algorithm to find the suitable values of *C* and *g*. Figure 7 is the prediction process of rotation errors based on IGWO-SVR, and the specific steps are as follows:

Step 1: Import five key manufacturing error factors X and rotation error Y into the prediction model as input and output data, perform normalization, and separate sample data into training and test sets.

Step 2: Set an optimization range of parameters about the SVR model, establish the rotation error prediction model based on SVR, and train the model using the training set data.

Step 3: Initialize the IGWO algorithm parameters, and generate the initial grey wolf population within the parameter range by following the OLHS method.

Step 4: Calculate the fitness (fitness value is mean square error) of each grey wolf by the SVR model, and save the top-three wolves of the best fitness as *wolfα*, *wolfβ*, and *wolfγ*.

Step 5: Update the IGWO algorithm parameters and *ω* wolves, and then calculate the fitness.

Step 6: Compare the fitness of each *ω* wolf with the corresponding fitness values of *wolfα*, *wolfβ*, and *wolfγ* in turn, and if it is better, replace the corresponding leading wolf’s position with the *ω* wolf’s position.

Step 7: Judge whether the current iteration count is equal to the maximum iteration count. If yes, output the bestC and bestg; otherwise, return to step 6.

Step 8: Replace C and g with bestC and bestg to establish an IGWO-SVR model of rotation errors.

Step 9: Test the rotation error prediction model with the test set data.

Step 10: Judge whether the rotation error prediction model’s forecast accuracy meets the assembly line requirement. If it meets the accuracy requirement, output the prediction results and save the prediction model; otherwise, go back to Step 3.

## 5. Result and Discussion

The prediction model in this paper was implemented by using Python 3.8 programming in Windows 10 system. This paper selects mean square error (MSE), mean relative error (MRE), and mean absolute error (MAE) as the evaluation metrics to assess the prediction performance of the IGWO-SVR model.

(25) $$MSE=\frac{1}{n}\overset{n}{\underset{i=1}{\sum }}{\left(Y_{i}-Y_{i}^{*}\right)}^{2}$$

(26) $$MAE=\frac{1}{n}\overset{n}{\underset{i=1}{\sum }}\left|Y_{i}-Y_{i}^{*}\right|$$

(27) $$MRE=\frac{\sum_{i=1}^{n}\frac{\left|Y_{i}-Y_{i}^{*}\right|}{Y_{i}}}{n}$$

where $Y_{i}$ denotes a real value of rotation error, $Y_{i}^{*}$ denotes a predicted value of rotation error, and n is the number of test samples.

### 5.1. Preprocessing of Data

In this paper, 100 sets of data from the actual production process of an RV reducer manufacturing company are selected as samples. Because different factors have different magnitudes, the sample data are normalized to eliminate the influence of magnitudes among factors. Then the sample data are divided into test samples and training samples at a rate of 2:8. A normalized mathematical expression is shown in Equation (28):

(28) $$x_{newi}=\frac{x_{i}-x_{min}}{x_{max}-x_{min}}$$

where $x_{newi}$ denotes the normalized value, $x_{i}$ denotes the raw value, and $x_{max}$ and $x_{min}$ denote the maximum value and the minimum value, respectively. Some of the normalized data are shown in Table 4.

### 5.2. Optimization Results of Parameters

In this paper, the parameters C and g in the SVR model need to be optimized. Therefore, the dimension of the optimized parameter in the IGWO algorithm is set to 2, and the value range of super parameters C and g is set as 0.01 to 100. Table 5 shows the values of the parameters of the algorithm.

**Figure 8:**
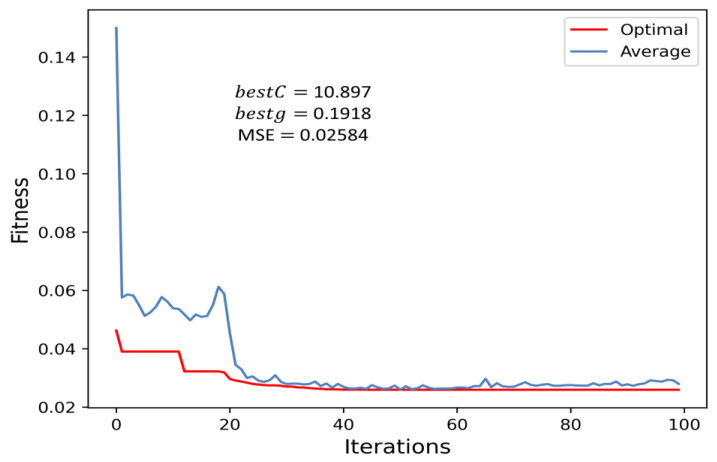
Fitness curves of different algorithm models.

The MSE is chosen as the fitness function, then the fitness of each individual is computed during the iterative process, and the results of the optimal parameters bestC and bestg are finally obtained. Finally, the bestC and bestg in SVR are 10.897 and 0.1918, respectively, by the IGWO algorithm, and the corresponding optimal fitness value at this point is 0.0258. The optimal fitness curve and average fitness curve in the iteration process are shown in Figure 8. As can be seen from Figure 8, the optimal fitness curve basically coincides with the average fitness curve after 20 iterations.

### 5.3. Analysis of Predictive Effect of the IGWO-SVR Model

**Figure 9:**
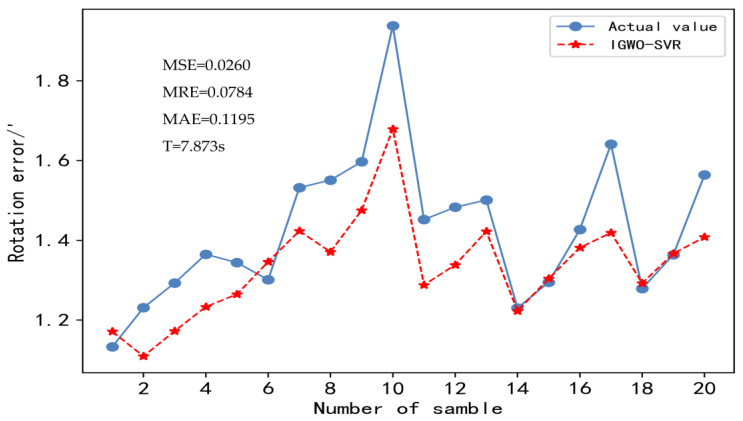
Prediction results of the IGWO-SVR model for rotation errors.

**Figure 10:**
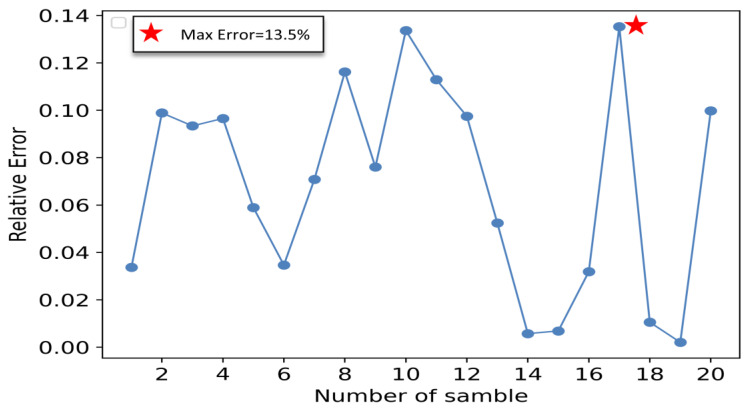
Relative error of the IGWO-SVR model for each individual sample point.

In this paper, MSE, MAE, MRE, and calculation time are used as criteria to evaluate the prediction effectiveness of the IGWO-SVR model, and as they get closer to 0, the better the prediction effectiveness of the model is. Figure 9 shows the prediction result of the model compared with the actual value, and Figure 10 shows the relative error for each sample point.

Figure 9 shows that the MSE, MRE, MAE, and calculation time of the IGWO-SVR model are 0.026, 0.0784, 0.1195, and 7.873s after 20 test set samples, respectively, indicating that the trained IGWO-SVR model achieves a high prediction accuracy and can also meet the requirement of enterprise production line beat in calculation time. Figure 10 shows that the maximum relative error of the forecasting model is 13.5%, which meets the enterprise’s requirement for the error to be within 20%. In summary, although the model has some errors, the overall prediction effect is good, it can more accurately express the nonlinear relationship between the rotation error and the dimensional errors of key parts, and its prediction accuracy and calculation time can meet the requirements of RV reducer enterprises.

### 5.4. Performance Evaluation of Model

In order to better evaluate the performance of the IGWO-SVR model, it is compared with the SVR models optimized by the PSO algorithm and the GWO algorithm, the existing BP neural network rotation error prediction method [6], and the SSA-BP neural network rotation error prediction method [7]. The parameters of different rotation error prediction models are shown in Table 6.

**Figure 11:**
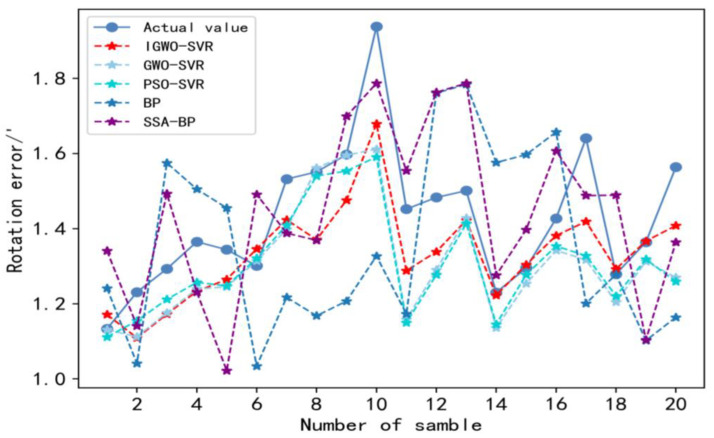
Prediction results of rotation error for different models.

Figure 11 and Table 7 clearly show the prediction results of different rotation error prediction methods. From Table 7, the MSE of IGWO is 0026, which is 27.37% lower than PSO-SVR method, 28.57% lower than GWO-SVR method, 78.53% lower than BP method, and 28.37% lower than SSA-BP method; the MRE of IGWO is 0.0784, which is 11.21% lower than PSO-SVR method, 13.94% lower than GWO-SVR method, 59.06% lower than BP method, and 37.68% lower than SSA-BP method; and the MAE of IGWO is 0.1195, which is 10.75% lower than PSO-SVR method, 12.65% lower than GWO-SVR method, 57.46% lower than BP method, and 32.52% lower than SSA-BP method. Therefore, the results of the data analysis show that the IGWO method is better than the other prediction methods at prediction accuracy. In addition, for calculations, the IGWO-SVR method outperforms the PSO-SVR model and significantly outperforms the BP and SSA-BP models. Although the computing efficiency of the IGWO-SVR method is a little slower compared with the GWO-SVR model, it can also meet the beat requirement of the RV reducer production line. Therefore, given the prediction accuracy and computational efficiency, the IGWO-SVR method has better prediction performance than the other methods.

**Figure 12:**
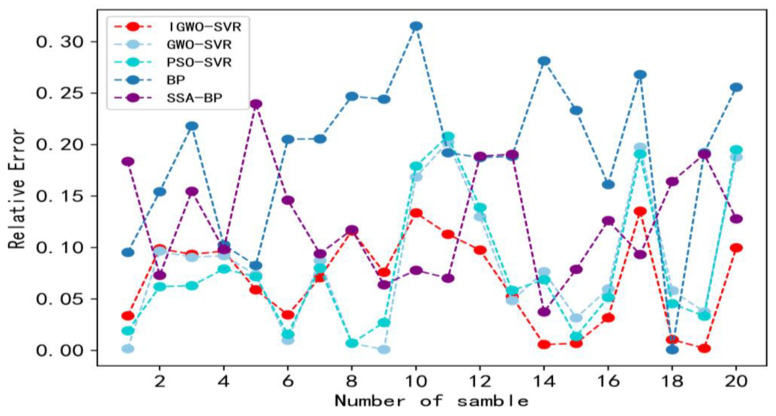
Relative errors of different models for rotation error prediction.

**Figure 13:**
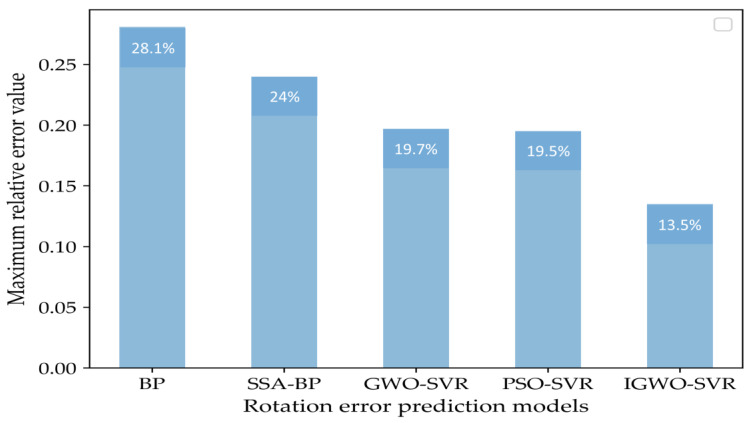
Maximum relative error of different prediction methods.

Figure 12 and Figure 13 show the relative error for each sample point and maximum relative error of different prediction methods. According to Figure 12, the relative error curve of the IGWO-SVR method is less volatile and more stable than those of the other methods. In addition, according to Figure 13, the maximum relative error of IGWO-SVR is 13.5%, which is not only within the 20% range required by companies but also lower than other forecasting methods. The maximum relative errors of GWO-SVR and PSO-SVR are 19.7% and 19.5%, respectively, and they are only slightly below 20%, so they are not as reliable. Additionally, the maximum relative errors of the BP neural network and the SSA-BP neural network are 28.1% and 24%, respectively, which can no longer meet the requirements of the forecasting accuracy of enterprises. Therefore, the prediction results of the IGWO-SVR method for rotation errors are more reliable than the other methods.

In summary, the IGWO-SVR method not only has good performance in prediction accuracy and prediction efficiency but also has low volatility and good stability. Thus, it can more accurately express the nonlinear relationship between rotation errors and the dimensional errors of key parts. Additionally, it provides a more accurate quality prediction input model for the part-matching model of the RV reducer, which will be more conducive to improving assembly quality.

## 6. Conclusions

This study solves the problem that the traditional rotation error research methods cannot be applied to the parts-matching process, because they are time-consuming and have poor calculation accuracy. Therefore, this paper proposes to use the SVR method, combined with the improvement of the grey wolf optimization algorithm, to predict the rotation error with high accuracy and speed. In addition, this paper takes the RV-40E reducer as a case to verify the performance of the method. The main contents include the following parts:

(1) Traditional GWO algorithm is enhanced based on the OLHS method, the cosine nonlinear convergence factor, and the dynamic weight strategy. Through verification, the IGWO algorithm has good optimization performance.
(2) The prediction model for the rotation error of the RV reducer based on IGWO-SVR is established by optimizing the *C* and g of SVR by using the IGWO algorithm. Additionally, its MSE is 0.026, running time is 7.843 s, and maximum relative error is 13.5%, which can meet the requirements of production beat and the product quality of enterprise.
(3) A comparison of the IGWO-SVR method with other methods shows that the former provides better prediction performance and the IGWO algorithm shows better parameter optimization performance.

The innovative contributions of this paper are the following three: (1) Improve its optimization performance by improving the traditional grey wolf optimization algorithm. (2) A new rotation error research method based on IGWO-SVR model is proposed for the disadvantages of existing rotation error research methods. (3) This research method can guide the assembly of RV reducers through the parts-matching process, thus improving its assembly quality and efficiency.

Although the IGWO-SVR method has good prediction performance for rotation errors, the method also has some drawbacks. First, the rotation error is affected by many factors, but the method considers only five key factors in the second-stage cycloidal pin-wheel transmission. Second, the method is only for the RV-40E model reducer, and the applicability to other models is not necessarily very high. Therefore, these issues will be considered in future work to obtain a more optimal prediction model for rotation errors.

## Figures and Tables

**Table 1:** Sensitivity index of manufacturing errors of key components.

Manufacturing Errors of Key Components	Index of Sensitivity	Weight %
Cycloid gear isometric modification error ($\theta_{1}$)	1.6131	23.040
Radius error of needle tooth center circle ($\theta_{2}$)	1.102	15.746
Cycloid gear shift modification error ($\theta_{3}$)	−1.1024	15.746
Needle tooth radius error ($\theta_{4}$)	−0.8065	11.519
Crankshaft eccentricity error ($\theta_{5}$)	0.00007	0.001
Accumulated pitch error of cycloidal gear ($\theta_{6}$)	−0.589	8.410
Needle hole circumferential position error ($\theta_{7}$)	0.587	8.341
Cycloid ring gear radial runout error ($\theta_{8}$)	0.201	2.871
Crank-bearing clearance ($\theta_{9}$)	1.000	14.283

**Table 2:** Test functions.

Test Functions	Dimension	Range	Min
$f_{1}=\overset{n}{\underset{i=1}{\sum }}x_{i}^{2}$	30	[−100, 100]	0
$f_{2}=\overset{n}{\underset{i=1}{\sum }}\left|x_{i}\right|+{П}_{i=1}^{n}\left|x_{i}\right|$	30	[−10, 10]	0
$f_{3}=\overset{n}{\underset{i=1}{\sum }}\left[x_{i}^{2}-10\cos\left(2\pi x_{i}\right)+10\right]$	30	[−5.12, 5.12]	0
$f_{4}=\frac{1}{4000}\overset{n}{\underset{i=1}{\sum }}x_{i}^{2}-{П}_{i=1}^{n}\cos\left(\frac{x_{i}}{\sqrt{i}}\right)+1$	30	[−600, 600]	0
$f_{5}=4x_{1}^{2}-2.1x_{1}^{4}+\frac{x_{1}^{6}}{3}+x_{1}x_{2}-4x_{2}^{2}+4x_{2}^{4}$	2	[−5, 5]	−1.0316
$f_{6}={\left(x_{2}-\frac{5.1}{4\pi^{2}}x_{1}^{2}+\frac{5}{\pi }x_{1}-6\right)}^{2}+10\left(1-\frac{1}{8\pi }\right)cosx_{1}+10$	2	[−5, 5]	0.3979

**Table 3:** Algorithm optimization results.

Function	Algorithm	Average	St.dev
$f_{1}$	PSO	3.73 × 10^−12^	5.45 × 10^−12^
	GWO	3.88 × 10^−48^	6.79 × 10^−48^
	SSA	2.76 × 10^−7^	6.27 × 10^−7^
	IGWO	1.69 × 10^−77^	1.97 × 10^−78^
$f_{2}$	PSO	1.59 × 10^−3^	1.84 × 10^−2^
	GWO	8.65 × 10^−45^	5.89 × 10^−44^
	SSA	5.54 × 10^−6^	1.59 × 10^−5^
	IGWO	4.07 × 10^−56^	1.43 × 10^−58^
$f_{3}$	PSO	3.67 × 10^−2^	5.32 × 10^−2^
	GWO	5.44 × 10^−15^	1.09 × 10^−16^
	SSA	7.98 × 10^−6^	2.06 × 10^−5^
	IGWO	0	0
$f_{4}$	PSO	0.0098	0.0105
	GWO	0.0025	0.0189
	SSA	3.75 × 10^−8^	9.42 × 10^−8^
	IGWO	0	0
$f_{5}$	PSO	−1.0316	4.66 × 10^−8^
	GWO	−1.0316	7.77 × 10^−8^
	SSA	−1.0316	7.54 × 10^−5^
	IGWO	−1.0316	3.57 × 10^−8^
$f_{6}$	PSO	0.3979	5.29 × 10^−7^
	GWO	0.3979	1.82 × 10^−8^
	SSA	0.3979	1.97 × 10^−4^
	IGWO	0.3979	1.83 × 10^−8^

**Table 4:** Partially normalized sample data.

Sample	$\theta_{1}$	$\theta_{2}$	$\theta_{3}$	$\theta_{4}$	$\theta_{5}$	Rotation Error (Y/′)
1	0.156	0.500	0.903	0.850	0.800	1.133
2	0.250	0.350	0.288	0.350	0.400	1.231
3	0.750	0.650	0.711	0.350	0.600	1.938
4	0.750	0.500	0.288	0.50	0.400	1.452
5	0.500	0.650	0.288	0.650	0.400	1.564
6	0.843	0.150	0.903	0.850	0.500	1.272
7	0.500	0.150	0.903	0.500	0.800	1.464
8	0.843	0.850	0.500	0.150	0.800	1.473
9	0.312	0.500	0.807	0.750	0.700	1.190
10	0.5	0.250	0.500	0.500	0.700	1.240

**Table 5:** Parameter values of the IGWO algorithm.

Number of Optimizations	Scope of Optimizations	Number of Wolves	Maximum Iterations	Mode Norm of Space
2	[0.01, 100]	20	100	10

**Table 6:** Parameter values for different rotation error prediction models.

Model	Parameter	Value
BP neural network	Learning rate	0.01
	optimizer	Stochastic gradient descent
SSA-BP neural network	Learning rate	0.01
	optimizer	Stochastic gradient descent
IGWO-SVR	bestC	10.897
	bestg	0.1918
GWO-SVR	bestC	1.275
	bestg	6.183
PSO-SVR	bestC	1.059
	bestg	7.532

**Table 7:** Prediction results of rotation error for different models.

Prediction Model	Evaluating Indicator			Time Duration/s
	MSE	MRE	MAE	
IGWO-SVR	0.0260	0.0784	0.1195	7.843
PSO-SVR	0.0358	0.0883	0.1339	8.926
GWO-SVR	0.0364	0.0911	0.1368	6.542
BP neural network	0.1211	0.1915	0.2809	10.508
SSA-BP neural network	0.0363	0.1258	0.1771	11.851

## Data Availability

The data presented in this study are available on request from the corresponding author. The data are not publicly available, because they involve corporate data privacy.
